# Maternal psychological stress-induced developmental disability, neonatal mortality and stillbirth in the offspring of Wistar albino rats

**DOI:** 10.1371/journal.pone.0171089

**Published:** 2017-02-21

**Authors:** Sakthivel Govindaraj, Annadurai Shanmuganathan, Ravindran Rajan

**Affiliations:** Department of Physiology, Dr. A.L.M Post Graduate Institute of Basic Medical Sciences, University of Madras, Taramani Campus, Chennai, India; Universitat de Barcelona, SPAIN

## Abstract

**Background:**

Stress is an inevitable part of life, and maternal stress during the gestational period has dramatic effects in the early programming of the physiology and behavior of offspring. The developmental period is crucial for the well-being of the offspring. Prenatal stress influences the developmental outcomes of the fetus, in part because the developing brain is particularly vulnerable to stress. The etiology of birth defects of the offspring is reported to be 30–40% genetic and 7–10% multifactorial, with the remaining 50% still unknown and also there is no clear cause for neonatal mortality and still-birth.

**Objective:**

The present study explores the association of maternal psychological stress on mother and the offspring’s incidence of birth defects, stillbirth, and neonatal mortality.

**Study design:**

Pregnant animals were restrained to induce psychological stress (3 times per day, 45 minutes per session). Except control group, other animals were exposed to restraint stress during the gestational period: early gestational stress (EGS, stress exposure during 1^st^ day to 10^th^ days of gestational period), late gestational stress (LGS, stress exposure during 11^th^ day to till parturition), and full term gestational stress (FGS, stress exposure to the whole gestational period). The effects of maternal stress on the mother and their offspring were analyzed.

**Results:**

Expectant female rats exposed to stress by physical restraint showed decreased body weight gain, food intake, and fecal pellet levels. Specifically, the offspring of female rats subjected to late gestational and full term gestational restraint stress showed more deleterious effects, such as physical impairment (LGS 24.44%, FGS 10%), neonatal mortality (EGS 2.56%, LGS 24.44%, FGS 17.5%), stillbirths (FGS 27.5%), low birth weight (EGS 5.42g, LGS 4.40g, FGS 4.12g), preterm births (EGS 539 Hrs, LGS 514 Hrs, FGS 520.6 Hrs), and delayed eyelid opening (EGS 15.16 Days, LGS 17 Days, FGS 17.67 Days).

**Conclusion:**

The results of this study reveal that maternal stress may be associated with the offspring’s abnormal structural phenotyping, preterm birth, stillbirth and neonatal mortality.

## Introduction

Maternal stress not only affects the mother, but also affects the developing fetus, and may have long-lasting effects, eventually affecting their subsequent progeny [1]. Stress exposure during the gestational period has potential adverse effects on the developing fetus, which may contribute to the early onset of many pathological conditions [2]. The developing fetal brain is more vulnerable to stress, which may cause abnormal phenotypes, both structurally and functionally [3]. Maternal stress has been shown to lead to birth defects [4], with both studies from animal models and human patients demonstrating stillbirth and infant mortality [5][6] in the offspring when exposed to stress during gestational period. In the present study, it has been observed that when female Wistar albino rats are exposed to restraint stress during their gestational period, their offspring have lower birth weights, and greater incidences of neonatal mortality, still birth, and birth defects.

Birth defects, neonatal mortality, and stillbirths are major socio-economic problems in developing and low income countries. About 7.9 million children are born with serious birth defects every year, constituting 6 percent of total births worldwide. These birth defects contribute to about 7% of neonatal mortality; furthermore, 3.3 million deaths each year are of children under five years of age [7]. The etiology of the birth defects by genetic and multifactorial causes has been estimated to be about 30–40% and 7–10%, respectively, but the cause for the remaining 50% remains to be understood [8].

One mechanism by which maternal stress affects the fetus is via the hypothalamic-pituitary-adrenal (HPA) axis and metabolic system. The developing or immature brain has a poor ability to respond and adapt to stress, which causes long-lasting, adverse effects throughout life by altering neuronal architecture [9]. Maternal stress causes an increased secretion of stress hormones, namely, glucocorticoids (specifically, cortisol in human and corticosterone in rodents) by disturbing the negative feedback of the HPA axis, which leads to an increase in glucocorticoid levels in the placenta. This disruption further activates the fetal HPA axis, thereby causing an increased level of circulating glucocorticoid in the fetus that affects the developing brain [10].

Additionally, maternal stress can harm the developing fetus by altering its metabolic state. For instance, increased glucocorticoid (GC) level can lower the mRNA expression levels of hypothalamic appetite regulatory peptides levels such as neuropeptide Y (NPY), agouti-related protein (AGRP), and cocaine and amphetamine-regulated transcript (CART), which causes a decrease in the food intake and thus body weight of the animals [11]. Increased blood GC levels also alter fetal metabolism by increasing lipolysis in skeletal muscle and adipose tissue, decreasing glucose uptake in muscle, increasing gluconeogenesis in the liver, and reducing protein synthesis in muscle [12].

This study is aimed to evaluate the association of maternal psychological stress with offspring birth defects, stillbirth, neonatal mortality and delayed eyelid opening. Restraint stress during the gestational period results in the alteration of normal fetal development.

## Materials and methods

### Animal model & animal maintenance

Adult female Wistar albino rats (*Rattus norvegicus*) with an average weight of 160 ± 20 g were used for the study. For breeding, healthy adult male Wistar albino rats were used. Each individual animal was observed for the welfare in the course of experiment and this study does not entail euthanasia. The neonatal mortality and/or stillbirth were expected as an outcome of this study and the same was reviewed by the Institutional Animal Ethics Committee and proper clearance was obtained (IAEC No: 01/01/2015) prior to the commencement of experiments; furthermore, experiments were conducted in accordance with the guidelines of Committee for the Purpose of Control and Supervision of Experiments on Animals (CPCSEA), India. The quarantine procedures were followed according to the recommendations of the Canadian Council Guide to the Care and Use of Experimental Animals (1993). Experimental animals were maintained under controlled conditions with respect to temperature (23 ± 2°C), humidity (50 ± 5%) and light (12h light/dark cycle) in the central animal house facility of our institution. The animals were fed *ad libitum* with a standard rat pellet diet and drinking water.

### Breeding of animals

Three female rats were allowed to mate with one fertile, sexually-active male rat for one night. The next day morning, the vaginal plug was collected from the females. A vaginal smear was prepared and examined under the microscope for the presence of sperm. Presence of sperm served as the confirmation of pregnancy; as such, that day was recorded as “day zero” of pregnancy. The pregnant rats were then housed individually in separate cages.

### Experimental groups

The pregnant animals were randomly divided into four groups, each of which consisted of six animals. Group I: control group, group II: Early Gestational Stress (EGS)—gestational animals that were exposed to stress by restraint from the 1^st^ day to 10^th^ day of the gestational period, group III: Late Gestational Stress (LGS)—gestational animals that were exposed to stress by restraint from the 11^th^ day of gestation to parturition and group IV: Full Term Gestational Stress (FGS)—gestational animals that were exposed to stress by restraint throughout the gestational period, i.e. from the 1^st^ day to parturition.

### Psychological stress model

The animal model of psychological stress uses physical restraint of the animals [13]. The pregnant rats were exposed to stress by restraint using a wire mesh restrainer cage, three times per day; each episode was 45 minutes in duration, with a two hour interval between each episode [14]. The time of day for restraining the animals was shifted randomly to avoid habituation of the animals. The restrainer cage is designed to restrict the movement of animals without any pain, discomfort, suffocation, and physical suffering.

### Maternal food intake levels

The daily food intake, representing the consumption of food over a 24-hour span, of each experimental animal was calculated by subtracting the final food amount from the initial food amount. The average food intake of all the experimental groups for the periods of the 1^st^ day to 10^th^ day of gestation, and 11^th^ day to parturition, were calculated and compared with each other.

### Maternal fecal pellets level

The number of fecal pellets from maternal animals was measured daily (bedding material was changed daily). The average number of fecal pellets of all the experimental groups for the period of the 1^st^ day to 10^th^ day of gestation, and 11^th^ day to parturition, was calculated and compared with each other.

### Maternal body weight gain

The weight of each animal of all the experimental groups was measured in grams, and the maternal body weight gain of each animal was calculated by subtracting the initial weight from the final weight of each animal. The maternal body weight gain of all the experimental groups for the 1^st^ day to 10^th^ day of gestation and the 11^th^ day to parturition were calculated and compared with each other.

### Gestational length

The number of days taken for parturition from the day of pregnancy is referred to as the gestational length. The gestational length of the animals of each group was noted and compared with each other.

### Stillbirth

The newborn pups were carefully examined after parturition and pups born dead were considered as stillbirths. The percentage of stillbirth was calculated as follows:

$$\text{The percentage of the stillbirths}=\frac{\text{Number of stillbirths}}{\text{Number of total births in group}}\;X\;100$$

### Neonatal mortality rate

Death of the newborn pups (still-born animals were excluded) within three days following parturition was noted as neonatal mortality. The percentage of neonatal mortality was calculated as follows:

$$\text{The percentage of the neonatal mortality}=\frac{\text{Number of neonatal mortality}}{\text{Number of total births in group}}\text{X }100$$

### Birth defects

Birth defects are any structural or physiological abnormality present at the time of birth that develop either before or during birth. The newborn pups were carefully inspected for birth defects and the percentage of birth defects was calculated as follows:

$$\text{The percentage of the birth defects}=\frac{\text{Number of birth defects}}{\text{Number of total births in group}}\text{X }100$$

### Body weight of newborn pups

The body weight of newborn pups in all experimental groups was measured both on the day of delivery and the 30^th^ postnatal day. The body weight of the newborn pups on the day of delivery was compared among all experimental groups. Likewise, the body weight of newborn pups on the 30^th^ postnatal day was also compared among all experimental groups.

### Eyelid opening

The eyelids of the newly-born pups are closed during partition; these pups were examined daily for the opening of their eyelids. The day of eyelid opening was noted for each animals of every experimental group.

### Statistical analysis

The data were analyzed by SPSS for windows statistical package (version 20.0, SPSS Institute Inc., Cary, North Carolina). One-way analysis of variance (ANOVA) and followed by Tukey’s multiple comparison was performed to determine the significance between the groups and data are expressed as mean ± standard error of mean (SEM). The significance level was fixed at p< 0.05, “a” refers to “compared to control”, “b” to “compared to EGS group”, “c” to “compared to LGS”, and “d” to “compared to FGS” during 1–10 days of the gestational period and for other parameters. Additionally, “w”, “x”, “y” and “z” corresponded to “compared to control”, “compared to EGS group”, “compared to LGS”, and “compared to FGS”, respectively, during days of 11 to till parturition.

## Results

### Maternal food intake levels during gestation

**Fig 1:**
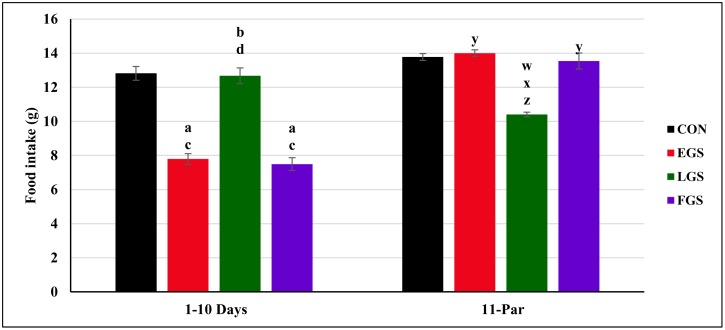
Maternal food intake during gestation. From one to 1^st^ day to 10^th^ day and 11^th^ to till parturition of control and experimental group namely, Early gestational stress (EGS), late gestational stress (LGS) and full term gestational group (FGS). The value p<0.05 are considered as significant.

The maternal food intake is shown in Fig 1. The maternal food intake level for the 1^st^ to 10^th^ day of gestation was compared among all four experimental groups, and there was a statistically significant difference between the groups [F(3,20) = 39.733]. Tukey post hoc test revealed that the EGS group showed a statistically significant decrease in maternal food intake for the 1^st^ day to 10^th^ day (7.79 ± 0.31g/day, p = 0.001) when compared to the control group (12.81 ± 0.40g/day, p = 0.001) and LGS group (12.67 ± 0.45g/day, p = 0.001). The maternal food intake level for the 1^st^ day to 10^th^ day for the LGS group was significantly increased when compared with the EGS and FGS groups (7.49g ± 0.37/day, p = 0.003). The FGS group showed decreased maternal food intake for the 1^st^ day to 10^th^ day and was significantly decreased, as compared to the control and LGS groups.

The maternal food intake level for the 11^th^ day to parturition was also compared among all four experimental groups and there was a statistically significant difference between the groups [F(3,20) = 36.437]. The EGS group showed significantly increased maternal food intake during the 11^th^ day to parturition (13.99 ± 0.19g/day, p = 0.001) as compared to LGS group (10.40 ± 0.13g/day, p = 0.001). The LGS group showed significantly decreased maternal food intake for the 11^th^ day to parturition as compared to the control (13.53 ± 0.24, p = 0.001), EGS, and FGS groups (12.70 ± 0.22g/day, p = 0.001). The maternal food intake for the 11^th^ day to parturition of the FGS group is significantly increased as compared with the LGS group.

### Maternal fecal pellets during gestation

**Fig 2:**
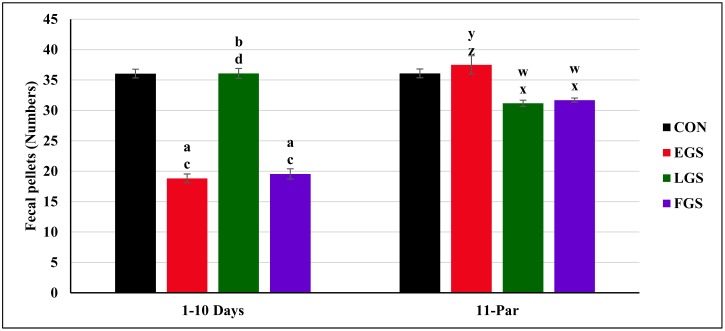
Maternal fecal pellets during gestation. From one to 1^st^ day to 10^th^ day and 11^th^ to till parturition of control and experimental group namely, Early gestational stress (EGS), late gestational stress (LGS) and full term gestational group (FGS). The value p<0.05 are considered as significant.

Fig 2 represents the data of maternal fecal pellets: the maternal fecal pellets for the 1^st^ day to 10^th^ day of gestation was compared among all experimental groups, and there was a statistically significant difference between the groups [F(3,20) = 157.004]. The group EGS (18.81 ± 0.73 pellets/day, p = 0.001) showed significantly decreased maternal fecal pellets for the 1^st^ to 10^th^ day of gestation, relative to the control (36.05 ± 0.72 pellets/day, p = 0.001) and LGS groups (36.08 ± 0.81 pellets/day, p = 0.004). Maternal fecal pellets of the1^st^ day to 10^th^ day of gestation of the LGS group was significantly increased as compared to the EGS and FGS groups (19.55 ± 0.84 pellets/day, p = 0.001). The number of maternal fecal pellets of the 1^st^ day to 10^th^ day of gestation of FGS group was significantly decreased relative to the control and LGS groups.

The number of maternal fecal pellets for the 11^th^ day to parturition was also compared among all four experimental group, and there was a statistically significant difference between the groups [F(3,20) = 12.853]. The EGS group showed significantly increased (37.49 ± 1.48 pellets/day, p = 0.001) numbers of fecal pellets for the 11^th^ day to parturition than the LGS (31.16 ± 0.51 pellets/day, p = 0.004) and FGS groups (31.68 ± 0.33 pellets/day, p = 0.010). The amount of maternal fecal pellets for the 11^th^ day to parturition of the LGS group was significantly decreased than the control (36.09 ± 0.71 pellets/day, p = 0.010) and EGS groups. The FGS group showed a decrease in maternal fecal pellets for the 11^th^ day to parturition, as compared to the control and EGS groups.

### Maternal body weight gain during the gestational period

**Fig 3:**
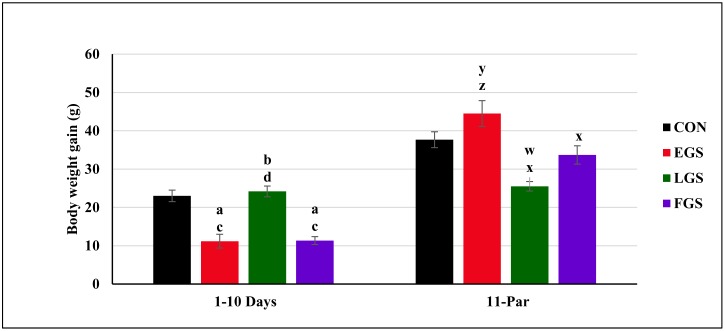
Maternal body weight gain during gestation. From one to 1^st^ day to 10^th^ day and 11^th^ to till parturition of control and experimental group namely, Early gestational stress (EGS), late gestational stress (LGS) and full term gestational group (FGS). The value p<0.05 are considered as significant.

Tukey post hoc multiple comparison shows that there was a statistically significant [F(3, 20) = 20.359], difference between the groups for maternal body weight gain for the 1^st^ day to 10^th^ day of gestation. Fig 3 shows the maternal body weight gain during the gestational period of control and stress-exposed groups during the 1^st^ to 10^th^ day of gestational period and 11^th^ day to parturition. The maternal body weight gain during 1^st^ to 10^th^ day of the gestational period was significantly decreased in the EGS group (11.16 ± 1.13g/1–10 days, p = 0.001) as compared to the control (23 ± 1.50g/1–10 days, p = 0.001) and LGS groups (24.16 ± 1.40g/1–10 days, p = 0.001). The LGS group showed a significantly increased maternal body weight gain of 1^st^ day to 10^th^ day of gestation as compared to the EGS and FGS groups (11.33 ± 1.02g/1–10 days, p = 0.001). The maternal body weight gain of FGS group was significantly decreased when compared to the control and LGS groups during 1^st^ day to 10^th^ day of gestation period.

The maternal body weight gain during the 11^th^ day to parturition was also compared among all four experimental groups, and there was a statistically significant difference between the groups [F(3,20) = 8.899]. The maternal body weight gain for the 11^th^ day to parturition of the EGS group (42.16 ± 3.35g/11-par, p = 0.001) was significantly increased compared to the LGS (25.5 ± 1.23g/11- par, p = 0.009) and FGS groups (33.66 ± 2.38g/11-par, p = 0.003). The LGS group showed decreased maternal body weight gain from the 11^th^ day to parturition, as compared to the control (37.66 ± 2.07g/11–22 days, p = 0.009) and EGS groups. The maternal body weight gain from the 11^th^ day to parturition was significantly lower in the FGS group than the EGS group.

### Gestational length

**Fig 4:**
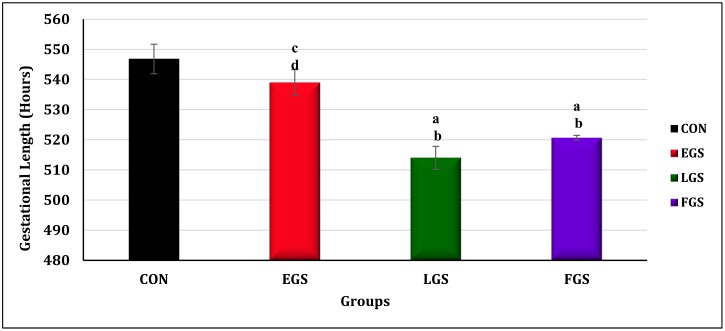
Gestational length. Early gestational stress (EGS), late gestational stress (LGS) and full term gestational group (FGS). The value p<0.05 are considered as significant.

**Fig 5:**
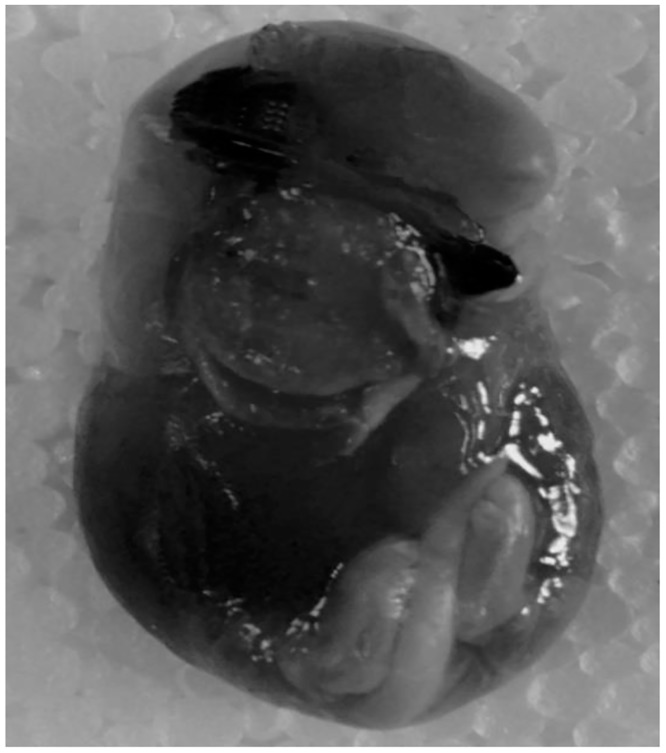
Preterm birth.

There was a statistically significant difference in gestational length among the experimental groups, as determined by one-way ANOVA [F(3,20) = 16.565]. The gestational length of the four groups and their statistical significance are given in Fig 4 and preterm birth is shown in Fig 5. The gestational length of the EGS group (539 ± 4.25hrs. p = 0.01) was significantly higher than the LGS (514 ± 3.80 hrs. p = 0.001) and FGS groups (520.67 ± 0.76hrs. p = 0.013). The LGS group showed a significantly decreased gestational length as compared to the control (546.83 ± 4.87hrs. p = 0.001) and EGS groups. The gestational period of the FGS group was significantly decreased as compared to the control and EGS groups. The EGS group also showed a slightly shorter gestational length, but this difference was not significant as compared to the control.

### Stillbirth

**Fig 6:**
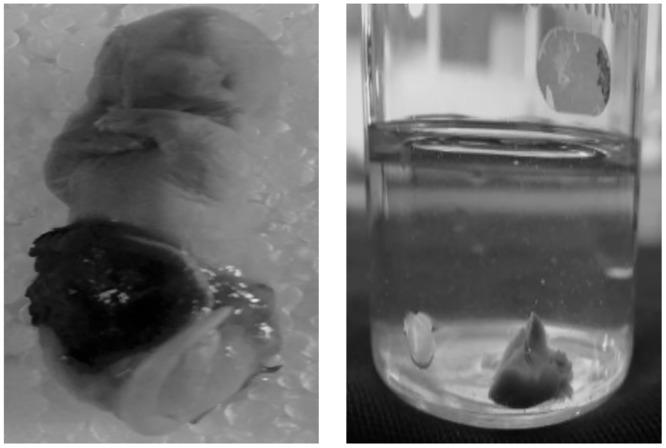
(A) Stillbirth and (B): Immature lung of stillbirth animal.

Stillbirths were observed only in the progeny of full term gestational stress-exposed animals (FGS groups) and the incidence was 27.5%. No stillbirths occurred in the control, EGS, or LGS groups. Table 1 and Fig 6 & 6A depict the stillbirths of the pups.

**Table 1:** Developmental anomalies. Neonatal mortality, stillbirth and birth defects of experimental groups.

	Experimental groups			
	CON	EGS	LGS	FGS
**Neonatal Mortality (%)**	0	2.56	24.44	17.5
**Stillbirth (%)**	0	0	0	27.5
**Birth Defects (%)**	0	0	24.44	10

### Neonatal mortality rate

**Fig 7:**
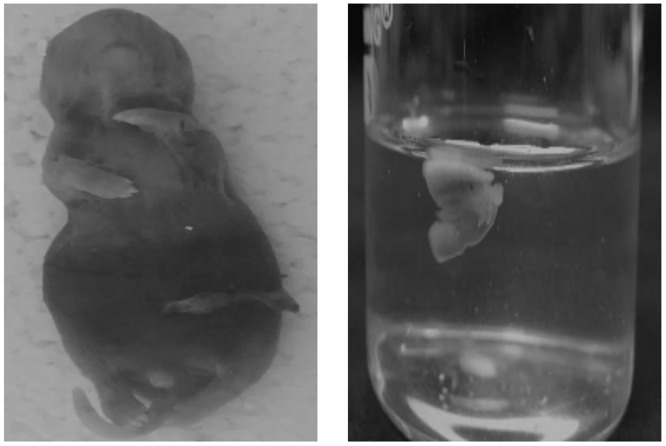
(A) Neonatal mortality and (B) Mature lung of neonatal mortality animal.

Neonatal mortality was observed in all the maternal stress-exposed groups, namely EGS, LGS and FGS. However, the mortality rate was higher (24.44%) in the LGS group as compared to the EGS (2.56%) and FGS (17.5%) groups. Neonatal mortality did not occur in the control group. Fig 7 & 7A and also Table 1 illustrate the neonatal mortality rate.

### Birth defects

**Fig 8:**
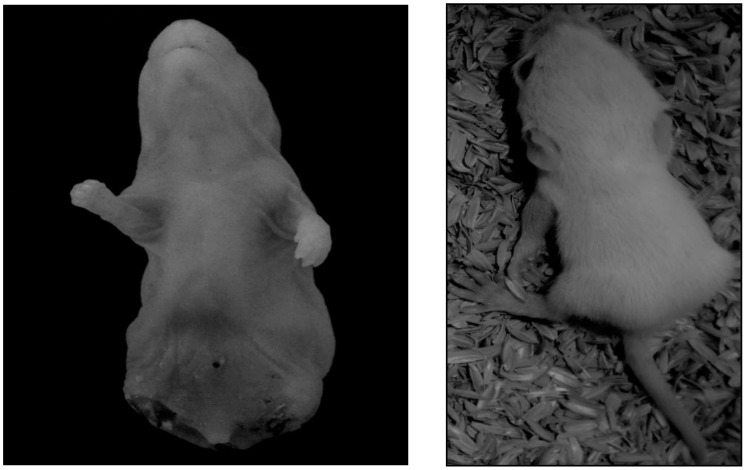
Birth defects.

There was no birth defects of any kind occurred in the offspring of the control or EGS groups. The percentage of birth defects in the LGS and FGS groups were 24.44% and 10%, respectively. Fig 8 and Table 1 depict the birth defects observed in the offspring of LGS and FGS groups.

### Body weight of the newborn pups

**Fig 9:**
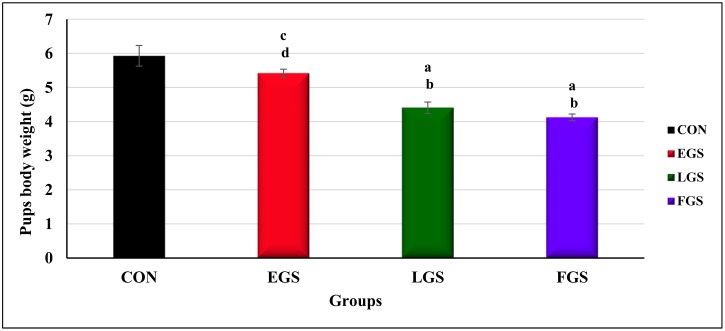
Body weight of the new born pups. Early gestational stress (EGS), late gestational stress (LGS) and full term gestational group (FGS). The value p<0.05 are considered as significant.

The body weight of the newborn pups on the 1^st^ day of parturition was significantly different between the experimental groups, as analyzed by one-way ANOVA [F(3,20) = 19.746]). The body weight of the newborn pups of the FGS group (4.12 ± 0.09g, p = 0.001) was significantly lower than either the control (5.93 ± 0.30g, p = 0.003) or EGS groups (5.42 ± 0.11g, p = 0.006). As shown in Fig 9, the LGS group (4.40 ± 0.16g, p = 0.006) showed a significantly decreased body weight of newborn pups, as compared to the control and EGS groups.

**Fig 10:**
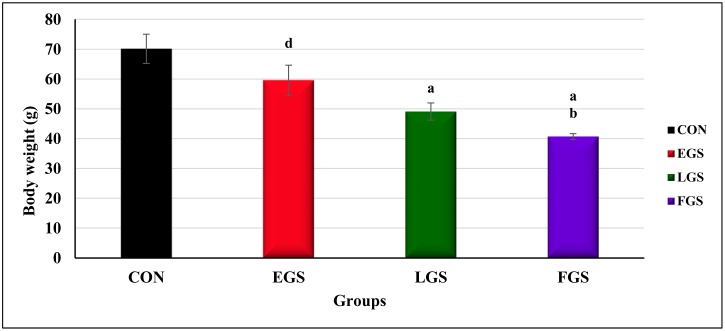
Offspring body weight on 30^th^ postnatal day. Early gestational stress (EGS), late gestational stress (LGS) and full term gestational group (FGS). The value p<0.05 are considered as significant.

The 30^th^ day of postnatal body weight of the offspring were also evaluated by one-way ANOVA [F(3,20) = 11.088]. The body weight of the offspring on the 30^th^ day of postnatal life in FGS group (40.68 ± 0.99g, p = 0.01) was significantly lower than in the control (70.15g ± 4.89, p = 0.005) and EGS groups (59.58 ± 5.06g, p = 0.01). As shown in Fig 10, the offspring in the LGS group (49.08 ± 2.89g, p = 0.005) showed a decrease in body weight compared to those in the control group.

### Eyelid opening

**Fig 11:**
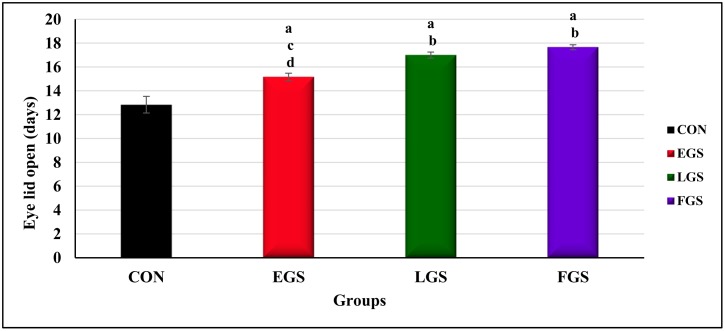
Eyelid open. Early gestational stress (EGS), late gestational stress (LGS) and full term gestational group (FGS). The value p<0.05 are considered as significant.

One way ANOVA analysis revealed a statistically significant difference in eyelid opening between the experimental groups [F(3,20) = 26.772] and the control group. The eyelid opening of the EGS group (15.16 ± 0.30 days, p = 0.02) showed a significant delay compared to the control group (12.83 ± 0.70 days, p = 0.004), but was still less delayed than the LGS (17 ± 0.25 days, p = 0.02) and FGS groups (17.66 ± 0.21 days, p = 0.02), as shown in Fig 11. The experimental groups LGS and FGS required significantly more time for eyelid opening than the control group.

## Discussion

As per general perception, dams will increase their food intake during the progression of pregnancy to meet the nutritional needs of the growing fetus. However, an extensive variation in the food intake of pregnant dams during gestation was observed in the present study. The maternal food intake of the control animals was increased when compared to stress-exposed groups from the 1^st^ day to 10^th^ day of gestational period. EGS and FGS groups (i.e., the groups exposed to stress by restraint the animal between the 1^st^ and 10^th^ day of gestation) showed decreased food intake, which may be due to increased GC levels. Previous studies reported that higher circulating levels of GC can positively stimulate gluconeogenesis and liver metabolism, thereby inhibiting food intake. The LGS group was not exposed to restraint-stress during the 1^st^ to 10^th^ day of gestational period, and accordingly showed increased food intake during that time. Additionally, the LGS and FGS groups, which were exposed to restraint stress from the 11^th^ day to parturition, showed decreased food intake, which may also be due to the increased gluconeogenesis process, liver metabolism and alterations in hypothalamic appetite hormone levels during stress [12]; in contrast, the EGS group showed increased food intake on late gestational period. It was further confirmed by De Vos *et al*. that subcutaneous administration of a pharmacological dose of hydrocortisone (1, 10 and 100 μg/g/day) causes a decrease in body weight and food consumption [15].

Since the food intake of stress exposed groups was lower due to glycogenolysis and gluconeogenesis, the number of fecal pellets deposited was also accordingly lower in restraint stress exposed groups. The EGS and FGS groups showed a decrease in egested fecal pellets in the 1^st^ to 10^th^ day of gestation compared to the control group, whereas the number of fecal pellets were increased in the LGS group. Restraint stress-exposed groups from the 11^th^ day to parturition, namely the LGS and FGS groups, exhibited a decreased number of fecal pellets, which may be due to decreased food intake during the late gestational period. On the other hand, the EGS group showed increased number of fecal pellets due to increased food intake those days. Elevated glucocorticoids levels have been associated with increased catabolic effect, which leads to increases the circulating amino acids, glucose and lipids in plasma which turn off hunger signal in the brain [16]. Thus, reduced food uptake was observed in the stress exposed group.

Although it is observed that, a general trend of increasing body weight in the pregnant dams due to fetal growth. The wide range of body weight gain among the dams may be due to differential food intake, their individual cortisol levels, and the number of fetuses conceived. In the present study, a positive correlation was observed between body weight gain and stress exposure. Restraint stress-exposed groups showed significantly reduced maternal body weight gain during gestation compared to the control group. During the 1^st^ to 10^th^ day of gestation, the EGS and FGS groups showed decreased maternal body weight gain due to the exposure to restraint stress, whereas the LGS group showed increased maternal weight gain. Restraint stress during gestation from the 11^th^ day to parturition (the groups LGS and FGS) led to decreased maternal weight gain; in contrast, the EGS group showed increased maternal weight gain. In general, an increased level of cortisol will be observed during gestation, which is necessary for the normal development and maturation of fetal organs, including the liver, lungs, and brain [17]. However, elevated levels of cortisol can have a deleterious effect on fetal development. Zakrzewska *et al*. reported that the administration of dexamethasone (0.025mg/kg) for three days induced a significant reduction in maternal body weight and food intake [18]; furthermore, animals exposed to maternal immobilization stress showed reduced maternal body weight gain during gestation [19].

An increased level of the hormone estrogen is necessary to stop ovulation to support the pregnancy, which results in increased cortisol in the plasma. The increased level of estrogen disturbs the cortisol catabolism in the liver [20]; hence, slightly elevated levels of estrogen were observed during pregnancy. This was further increased when animals were exposed to stress during the pregnancy, influencing the onset of delivery [17]. This abruptly increased levels of cortisol, resulting in preterm birth. In the present study, when compared to the control animals, the restraint stress-exposed animals (the LGS and FGS groups) showed both a significantly higher incidence of preterm birth and shorter gestational length, while the EGS group showed no difference in either. The higher incidence of preterm birth and shorter gestational length in the LGS and FGS groups may be due to an increased level of glucocorticoids. Some clinical studies also reported that in utero exposure to synthetic glucocorticoids can cause preterm births [20]; additionally, both increased placental CRH concentration [21] and adverse childhood experience [22] are associated with preterm birth.

Stillbirth is a major cause for neonatal mortality globally, and two-thirds of stillbirths occur in Southeast Asia. However, the cause of stillbirth is still not fully understood. Studies in humans have reported that people exposed to psychological stress during their pregnancy showed a greater incidence of stillbirths. There are several mechanisms for stress-induced stillbirth: i) an increased release of catecholamines, which limits the substrates delivered to the fetus, ii) the elevated level of glucocorticoid secretion, altering fetal cardiovascular and metabolic function, and iii) the suppression of appetite [23]. In the current study, the FGS group alone showed stillbirth, which may be due to the restraint stress exposure for the entire gestational period, causing the fetus to be exposed to higher glucocorticoid levels throughout the period of gestation, resulting in stillbirth. The WHO reported in 2015 that there were 2.6 million stillbirths globally, with more than 7,178 deaths a day, and that maternal hypertension was one of the causes for stillbirth [24][25]; indeed, stillbirth is directly correlated with maternal healthcare. Many factors may contribute to the incidence of stillbirth, including an increased GC level, followed by an inhibition of glucose uptake by the muscle, muscle wasting/atrophy, the suppression of protein synthesis while promoting protein degradation and amino acid export [26], higher glucocorticoids level affecting lung structure [27] and altering lung maturation [28].

Neonatal mortality is also associated with maternal stress exposure during pregnancy; with this in mind, we noted that neonatal mortality was observed in the EGS, LGS and FGS groups. Among these groups, the LGS and FGS groups showed increased mortality rate, which may be due to increased GC level during embryogenesis, followed by defects in organogenesis, which is a crucial period for fetal development. Indeed, GC plays a vital role in fetal organogenesis, especially lung maturation, liver and brain [29], dendrite and axonal terminal maturation. Though slightly increased levels of glucocorticoid are necessary for the development and maturation of fetal organ, an abrupt increase in these hormones can have deleterious effects on fetal postnatal survival. However, persisting increased GC levels may cause aberrant organ development. In the present study, we have reported immature lung development resulting from restrain stress, and this may be account for increased neonatal mortality. This is supported by the findings of a clinical study, which reported that violence against women increases the risk of infant and child mortality [30]. People who experienced stress before the conception period have been shown to be at a greater risk for infant mortality [31].

Birth defects are a major socioeconomic problem in developing countries, the causes and mechanisms of which are not fully understood. From the present study, we conclude that maternal prenatal stress is one causative factor, as animals exposed to prenatal maternal stress were at greater risk of offspring birth defects. The LGS and FGS groups showed increased offspring birth defects; specifically, the LGS group showed increased birth defects as compared to other groups, with an almost 2.5-fold higher incidence of birth defects in the offspring (24.44%) as compared to the FGS group (10%), which may be due to fact that most organogenesis happens between 10–22 days of the gestational period. In the LGS group, it is assumed that an elevated GC level during this period may cause inhibition of glucose uptake in muscle, decreased protein synthesis, protein degradation, muscle atrophy [12] and inhibition of calcium uptake for bone formation [32], all of which may contribute to the risk of birth defects. Earlier studies reported that maternal corticosteroids exposure increased the incidence of birth defects [33], and some clinical studies also reported that maternal stress may be a risk factor for birth defects [4].

Among the stress-exposed groups, the FGS and LGS exposed groups showed a significant decrease in the offspring’s birth weight, which may also be due to elevated GC level, ultimately causing decreased protein synthesis and muscle breakdown during development [34]. NR3C1 is the GC receptor and is involved in cell proliferation and differentiation; its degree of methylation, and thus gene activity, may be influenced by newborn birth weight [35] and several clinical studies have reported that increased methylation status of 11-β-HSD gene is associated with low birth weight [36]. It has been reported that maternal immobilization stress is associated with decreased birth weight [19]. Maternal stress affects postnatal development and growth by altering liver and skeletal muscle metabolism. Earlier studies reported that excess GC exposure in the perinatal period can program the liver and skeletal muscle metabolism toward a poor metabolic phenotype in later life [37], probably because of upregulated GR expression and GC sensitivity in the visceral fat and liver [37]. The present study has reported that the LGS and FGS exposed groups showed decreased body weight on the 30^th^ postnatal day, further confirming that by maternal immobilization, stress-induced rats showed decreased body weight as compared to control offspring [38][39].

The delayed eyelid opening may indicate a delay or alteration in the development of the brain. Our present result indicates that stress can induce a delay in eyelid opening, as the LGS and FGS groups took more time for eyelid opening compared to other groups; this may be due to an alteration in the brain circuit by an elevated GC level. Delayed eyelid opening is also linked with disrupt auditory processing [40].

## Conclusion

The developing fetus is highly sensitive to adverse effects experienced by the mother, as the developing fetal brain is more plastic and has a poor ability to respond and adapt to maternal stress. Maternal stress affects the fetus by elevating the secretion of glucocorticoids and increasing the placental GC levels, which may lead to the stimulation of gluconeogenesis and the inhibition of glucose uptake by peripheral tissues, decrease in protein synthesis, increase in muscle wasting and the inhibition of calcium intake for bone formation, all of which may cause a decrease in maternal food intake, fecal pellets levels, and maternal body weight gain during the gestation period. Therefore, the elevation of glucocorticoids in maternal restraint stress may cause stillbirth, neonatal mortality, birth defects, decreased birth weight, postnatal development and delayed eyelid opening. Further studies are warranted to understand the underlying molecular mechanisms and structural and functional analysis of the gestational restraint stress induced adverse effects. The present study shows maternal stress exposure during gestational period has long term deleterious effects on the fetus development.
